# Synergistic effects of enzyme-microbe co-fermentation on the nutritional quality and digestibility of corn stalks

**DOI:** 10.3389/fmicb.2025.1721614

**Published:** 2026-01-05

**Authors:** Jianwei Wei, Meng Sun, Chunqiang Wang, Qingyue Wang, Yingying Huang, Wei Ma, Ying Wang

**Affiliations:** 1 Department of Animal Science, College of Agriculture, Jinzhou Medical University, Jinzhou, Liaoning, China; 2 Liaoning Provincial Key Laboratory of Animal Product Quality and Safety, Jinzhou Medical University, Jinzhou, Liaoning, China

**Keywords:** probiotics, lignocellulolytic enzymes, corn stover, *in vitro* digestibility, ruminant production

## Abstract

**Introduction:**

This study aims to investigate the synergistic effects of combined probiotics and composite cellulase on the fermentation quality, nutritional value, and *in vitro* digestibility of corn stover.

**Methods:**

The experiment was designed with different addition methods of combined probiotics and enzyme preparations to establish a control group (CON), a probiotics-only group (J), an enzyme-only group (M), and a combined enzyme-probiotics group (MJ), with three replicates per group. The dynamic changes in the fermentation quality of corn stover were measured at 7, 14, 21, and 28 days of mixed fermentation. Additionally, *in vitro* simulated rumen fermentation technology and principal component analysis were employed to evaluate the rumen fermentation characteristics of mixed silage at 7, 14, 21, 28 days and after 72 h, and the feasibility of its feeding was confirmed by slaughtering Yanbian yellow cattle following feeding trials to assess production performance.

**Results:**

The results showed that the MJ group exhibited the best fermentation quality compared to the CON, M, and J groups, with significantly reduced pH and ammonia nitrogen levels (*p* < 0.05), and significantly increased lactic acid and acetic acid contents (*p* < 0.05). Neutral detergent fiber, acid detergent fiber, and lignin significantly decreased (*p* < 0.05), while crude protein retention increased and dry matter degradation decreased. *In vitro* rumen fermentation further indicated that the MJ group had significantly higher DM digestibility compared to the CON group, and CP and fiber digestibility were significantly higher than in the CON, M, and J groups (*p* < 0.05), with total volatile fatty acid production significantly higher than in the CON, M, and J groups. Principal component analysis results showed that the MJ group remained closely associated with nutritional indicators over time. Feeding trials confirmed that the MJ group significantly improved the average daily gain, feed conversion efficiency, and slaughter performance of Yanbian yellow cattle compared to the CON group (*p* < 0.05).

**Discussion:**

The results indicate that combined enzyme-probiotic treatment can synergistically enhance the feeding value of corn stover and the production performance of ruminants, providing a feasible technical pathway for the efficient utilization of agricultural waste.

## 1 Introduction

The main component of corn stover is lignocellulose, which includes cellulose, hemicellulose, and lignin. The structure of woody cellulose is complex and difficult to degrade efficiently (Mosier et al., 2005). In particular, lignin forms a dense natural barrier through covalent bonds with hemicellulose, which is resistant to degradation by rumen microorganisms, thereby becoming a key limiting factor in the utilization of straw as feed (Li et al., 2016).

To overcome this barrier, enzymatic treatment and microbial fermentation are widely used due to their respective characteristics. Enzymatic treatment, using enzymes such as lignin peroxidase, manganese peroxidase, laccase, and cellulase, can efficiently and specifically catalyze lignin depolymerization and cellulose hydrolysis, directly breaking down their complex structures with rapid action (Chukwuma et al., 2020). However, its industrial application is often limited by high costs, sensitivity to reaction conditions, and poor permeability in solid substrates (Kristensen et al., 2009). On the other hand, microbial fermentation, using organisms such as white-rot fungi and *Bacillus species*, can self-replicate and continuously secrete various enzymes, physically penetrate substrates through mycelia, and metabolically utilize the degradation products, thereby avoiding feedback inhibition (Sigoillot et al., 2012). Nevertheless, microbial fermentation has a relatively long cycle and requires strict cultivation conditions (Zhao et al., 2018). Given the inherent limitations of both enzymatic treatment and microbial fermentation, combining the two can create complementary advantages: enzymes provide immediate and efficient initial degradation, while microorganisms ensure sustained and stable subsequent conversion, thereby synergistically enhancing overall degradation efficiency. In recent years, enzyme–bacterium complex fermentation technology has garnered widespread attention in the field of lignocellulose degradation and conversion. Numerous studies have employed various methods to explore its mechanisms. For instance, some researchers have used mixed microbial solid-state fermentation to induce the production of multi-enzyme complexes (e.g., co-culture of *Penicillium* and *Neurospora crassa*) to enhance the enzymatic saccharification efficiency of substrates such as poplar wood (Zhao et al., 2018). Other studies have achieved degradation rates of 67.0, 60.4, and 33.0% for lignin, cellulose, and hemicellulose, respectively, in corn stover using solid-state fermentation with *Phanerochaete chrysosporium* combined with exogenous cellulase (Wan and Li, 2010). Additionally, the combination of commercial enzymes and fermented crude enzyme extracts has been shown to increase enzymatic hydrolysis efficiency to over 88%, with an 80% substitution rate for commercial enzymes (Liu et al., 2013).

Among these approaches, enzyme–bacterium complex fermentation technology, which involves the exogenous addition of lignocellulolytic enzymes and specific probiotics to synergistically degrade fibrous structures and improve fermentation quality, has become an important direction for the resource utilization of agricultural waste (Yildirim et al., 2020). Recent studies have demonstrated that biological lignin degradation methods can significantly enhance the fermentation efficiency of rice straw, reduce greenhouse gas emissions, and improve its value as ruminant feed (Nazar et al., 2025b). For example, the Forage Processing and Integrated Livestock-Grassland Innovation Team at the Jiangsu Academy of Agricultural Sciences used laccase to degrade lignin in rice straw, finding that laccase effectively breaks down lignin, reduces cellulose crystallinity, and improves the hydrolysis efficiency of cellulase (Nazar et al., 2025a). Moreover, the combined addition of *lactic acid bacteria*, cellulase, and laccase significantly enhanced the silage fermentation quality of rice straw, preserving more water-soluble carbohydrates, lowering pH, and inhibiting the growth of harmful microorganisms (Li et al., 2024). These findings indicate that biological lignin degradation methods can convert rice straw into high-quality ruminant feed, reduce greenhouse gas emissions, and promote the reuse of agricultural waste (Chilakamarry et al., 2022).

However, existing research has predominantly focused on short-term changes in fermentation parameters, with a lack of systematic evaluation of ruminant growth performance and slaughter traits, which limits the broader application of this technology. Although there are numerous reports on microbial community responses and initial degradation effects during short-term fermentation, the continuous dynamic degradation patterns of lignocellulosic components during long-term fermentation, the evolution of key structures, and their systematic impact on final fermentation quality and feeding value remain insufficiently explored.

Based on the laboratory screening and determination of the types and ratios of probiotics, this study constructs a composite probiotic. to conduct a 28-day long-term fermentation experiment. By systematically investigating the synergistic enhancement effects of long-term enzyme–bacterium complex fermentation on the degradation mechanism of lignocellulose, fermentation quality, and feeding value of corn stover, this research not only deepens the understanding of material structural evolution and microbe–enzyme interactions during biological pretreatment but also provides important theoretical and practical foundations for the efficient utilization of straw resources as feed. The findings hold significant scientific value and application prospects for promoting the resource utilization of agricultural waste, alleviating feed supply pressure, and supporting the sustainable development of animal husbandry.

## 2 Materials and methods

### 2.1 Preliminary screening of enzyme formulation ratios

The enzyme treatment experiment on corn straw involved a total of nine treatments, each with three replicates as shown in Table 1. The fermentation substrate for each group was 500 g, with moisture adjusted to 65%, and 15 mL of molasses added. The enzymes required for each treatment were dissolved in 325 mL of distilled water and thoroughly mixed, then evenly sprayed onto the corn straw. The mixed corn straw was then transferred to one-way degassing fermentation bags (40 × 50 cm, material: food-grade nylon seven-layer co-extruded film PA/PE, thickness 0.18 mm) and vacuum sealed using a vacuum packaging machine.

**TABLE 1:** Preliminary screening of different enzyme preparations and their dosages.

Group	Cellulase	Xylanase	Lignin peroxidase	Manganese peroxidase	Laccase
1	–	–	–	–	–
2	0.50 g	0.50 g	–	–	–
3	0.33 g	0.33 g	0.33 g	–	–
4	0.33 g	0.33 g	–	0.33 g	–
5	0.33 g	0.33 g	–	–	0.33 g
6	0.25 g	0.25 g	0.25 g	0.25 g	–
7	0.25 g	0.25 g	0.25 g	–	0.25 g
8	0.25 g	0.25 g	–	0.25 g	0.25 g
9	0.20 g	0.20 g	0.20 g	0.20 g	0.20 g

The enzyme preparation was dissolved in distilled water and evenly sprayed onto the surface of corn stalks (CS) using a micro-sprayer. After thorough mixing, the mixture was transferred into fermentation bags (specifications: 40 × 50 cm; material: seven-layer co-extruded food-grade nylon PA/PE film, thickness 0.18 mm; product number: BYB086), which were purchased from Zhengzhou Baiyibao Biotechnology Co., Ltd.

### 2.2 Preparation of corn stover

Corn stover was obtained from Chaoyangchuan Town, Yanji City, China. After grain maturation, the stover was harvested, air-dried to a moisture content below 30%, and chopped into 3–5 cm pieces. The experimental design incorporated factors including treatment type and fermentation duration. As summarized in Table 2, a total of 16 treatment conditions were evaluated, each with three replicate fermentation bags. The treatments included: CON (control group), M (enzyme preparation group), J (composite probiotic group), and MJ (combined enzyme–probiotic inoculum group). The samples were subjected to four different fermentation periods denoted as A1, A2, A3, and A4, corresponding to 7, 14, 21, and 28 days, respectively.

**TABLE 2:** Treatment of corn straw with different enzyme bacterial preparations.

Test group	Treatment of samples	Fermentation time			
		A1	A2	A3	A4
CON	500 g of CS with 325 ml distilled water, and 15 ml molasses	CON fermented for 7 days	CON fermented for 14 days	CON fermented for 21 days	CON fermented for 28 days
M	500 g of CS mixed with 325 mL distilled water, 15 mL molasses and 1 g enzymes (0.2% cellulase, xyloglucosidase, lignin peroxidase, manganese peroxidase and laccase = 1:1:1:1:1) lignin peroxidase, lignin peroxidase enzyme activity > 0.1 U/mg, other enzyme activity > 20 U/mg)	M fermented for 7 days	M fermented for 14 days	M fermented for 21 days	M fermented for 28 days
J	500 g of CS was mixed with 325 ml distilled water, 15 ml molasses, and 10 ml of the bacterial composite (*Lactobacillus*: *Saccharomyces cerevisiae*: *Bacillus subtilis* in 3:2:1 proportion; the concentrations of the bacteria used for inoculation in this experiment were all 1 × 10^8^ CFU/mL).	J fermented for 7 days	J fermented for 14 days	J fermented for 21 days	J fermented for 28 days
MJ	500 g of CS was mixed with 325 mL distilled water, 15 ml molasses, 1 g of enzyme preparation, and 10 ml complex bacterial preparation	MJ fermented for 7 days	MJ fermented for 14 days	MJ fermented for 21 days	MJ fermented for 28 days

The additives were dissolved in distilled water and sprayed onto CS using a microsprayer. After mixing, the mixture was transferred to a fermentation bag (40 × 50 cm, material: food-grade nylon seven-layer co-extruded film PA/PE, thickness 0.18 mm) purchased from Zhengzhou Baiyibao Biotechnology Co., Ltd. (item No. BYB086).

### 2.3 Strain source and culture methods

The *Saccharomyces cerevisiae* (CICC 138631), *Bacillus subtilis* (CICC 10089), and *Lactobacillus* spp. (CICC 137997) used in this study were all obtained from the China General Microbiological Culture Collection Center (CGMCC) and stored at 4°C. Each strain was cultured using specific media, as shown in Table 3.

**TABLE 3:** Components of microbial culture media.

Name (active)	Source	strain number	Medium composition/L
*Saccharomyces cerevisiae*(3 × 10^8^ CFU/mL)	Kept in lab 4°C refrigerator storage	CICC 138631	5 g of potato starch, 20 g of glucose, 0.1 g of chloramphenicol
*Bacillus subtilis*(2 × 10^8^ CFU/mL)	Kept in lab 4 °C refrigerator storage	CICC 10089	10 g of peptone, 10 g of beef meal, 5 g of NaCl, 20 g of agar
*Lactobacilli*(6 × 10^8^ CFU/mL)	Kept in lab 4 °C refrigerator storage	CICC137997	10 g of peptone, 10 g of beef meal, 5 g of yeast extract, 2 g of dipotassium hydrogen phosphate, 2 g of diammonium citrate, 5 g of sodium acetate, 20 g of dextrose, 1.0 mL of Tween 80, 0.5 g of magnesium sulfate, 0.25 g of manganese sulfate

All the above media were autoclaved before use at 121 °C for 20 min at the time of use. All microbial strains were obtained from the China General Microbiological Culture Collection Center (CGMCC).

### 2.4 Chemical composition analysis

#### 2.4.1 Fermentation quality analysis

A 20 g fermented sample was mixed with 180 mL distilled water and incubated at 4°C for 24 h. After filtration through four layers of cheesecloth, fermentation indicators were measured. pH was determined using a pH meter (PHSJ-3F, CANY, Shanghai, China). The DLG score was calculated based on pH and volatile fatty acid (VFA) concentration and graded as follows: excellent (20–18), good (17–14), moderate (13–10), low (9–5), and spoiled (4–0). Concentrations of lactic, acetic, propionic, and butyric acids were measured via high-performance liquid chromatography (HPLC). Ammonia nitrogen (NH_3_-N) was determined by the phenol-hypochlorite colorimetric method (Broderick and Kang, 1980).

#### 2.4.2 Chemical composition analysis

Samples were dried at 65°C for 48 h to determine dry matter (DM). Crude protein (CP) was analyzed using a Kjeldahl nitrogen analyzer (Foss, 2300 Autoanalyzer, FOSS Analytical AB, Höganäs, Sweden), and the ratio of ammonia nitrogen to total nitrogen (AN/TN) was calculated. Neutral detergent fiber (NDF), acid detergent fiber (ADF), and acid detergent lignin (ADL) were determined using a fiber analyzer (ANKOM) and the Van Soest method (Van Soest et al., 1991). Ash-free NDF (NDFom) was obtained by igniting NDF residue at 535°C for 2 h. Hemicellulose content was calculated as the difference between NDF and ADF, and cellulose as the difference between ADF and ADL.

The loss rates of cellulose, hemicellulose, and lignin were calculated as: (W_0_ – W_1_)/W_0_ × 100%, where W_0_ and W_1_ represent the mass (g) before and after treatment, respectively (Zhao et al., 2020).

### 2.5 *In vitro* rumen fermentation

The experimental procedures were approved by the Ethics Committee of Yanbian University (Yanji, China; Approval No. 201908027). For each sample (on a dry matter basis), 1 g was weighed in triplicate into filter bags (F57; ANKOM Technologies, Macedon, NY, United States) and placed in 100 mL graduated glass incubation tubes. Rumen fluid was collected via rumen cannula from three Yanbian yellow cattle before morning feeding, mixed, and filtered through four layers of cheesecloth. The filtered fluid was combined with pre-warmed buffer at a 1:2 (v/v) ratio. Then, 70 mL of the mixture was added to each tube and incubated at 39°C in a shaking water bath for 72 h. The entire *in vitro* incubation process was repeated three times, each with duplicate samples.

After incubation, 5 mL of supernatant was collected for analysis of volatile fatty acids (VFA) and ammonia nitrogen (NH_3_-N). VFA concentrations were analyzed using gas chromatography (GC-2014; Shimadzu, Kyoto, Japan) equipped with a flame ionization detector and a capillary column (Rtx-Wax, 0.25 mm inner diameter, 0.25 μm film thickness; Restek, France) (He et al., 2017). The remaining supernatant was used for pH measurement. To determine apparent *in vitro* dry matter digestibility, post-incubation samples were rinsed with cold water, dried at 65°C for 48 h, and analyzed for *in vitro* crude protein digestibility (IVCPFD), *in vitro* neutral detergent fiber digestibility (IVNDFD), and *in vitro* acid detergent fiber digestibility (IVADFD) (Yi et al., 2023; Yoo et al., 2020). Digestibility of dry matter, NDF, ADF, and CP was calculated as: (initial weight - residual weight)/initial weight. All experiments were performed in triplicate to minimize error.

### 2.6 Growth performance and slaughter traits

#### 2.6.1 Experimental animals and grouping

Twenty-four healthy Yanbian yellow cattle steers with similar initial body weight (450 ± 20 kg) and age (18 months) were randomly assigned to two groups. The control group (CON, *n* = 12) received a basal diet, while the treatment group (*n* = 12) received the basal diet supplemented with 15% enzyme-probiotic fermented feed (replacing an equivalent amount of roughage). A 14-day adaptation period was implemented for environmental acclimation, diet adjustment, deworming, and vaccination. Initial body weight (IBW) was recorded before grouping.

### 2.6.2 Experimental diets

The basal diet was formulated according to the nutritional requirements of Yanbian yellow cattle (referencing NRC or Chinese beef cattle feeding standards), with an appropriate concentrate-to-forage ratio. It consisted of concentrate supplements (e.g., corn, soybean meal, wheat bran, premix) and roughage (e.g., corn silage, Leymus chinensis, or rice straw). Major nutritional indices (e.g., ME, CP, Ca, P) did not differ significantly between the two groups.

#### 2.6.3 Husbandry management

The trial lasted 90 days. Cattle were group-housed by treatment, with free access to feed and water. Feeding occurred at 5:00 and 16:00 daily. Feed offered and leftovers were recorded to calculate average daily feed intake (ADFI). Pens were regularly cleaned to maintain hygiene.

#### 2.6.4 Data recording

Initial body weight (IBW): Average of two consecutive mornings of fasting weight after the adaptation period.
Final body weight (FBW): Average of two consecutive mornings of fasting weight at the end of the trial.
Average daily gain (ADG): ADG = (FBW – IBW)/trial days.
Average daily feed intake (ADFI): ADFI = (total feed offered - total leftovers)/(trial days × number of cattle) (unit: kg DM/head/day).
Feed-to-gain ratio (F/G): F/G = ADFI / ADG (unit: kg DM/kg gain).

#### 2.6.5 Slaughter traits

At the end of the trial, six cattle from each group were randomly selected for slaughter in compliance with animal welfare and slaughter regulations.
Slaughter body weight (SBW): Weight after 24-h fasting and 2-h water deprivation.
Carcass weight (CW): Weight after bleeding, and removal of head, feet, hide, and internal organs (excluding kidneys and perirenal fat).
Dressing rate (DR): DR (%) = (CW/SBW) × 100.
Net meat weight (NMW): Total meat weight after deboning (including kidneys and perirenal fat).
Net meat percentage (NMR): NMR (%) = (NMW/SBW) × 100.
Meat-to-bone ratio: NMW/bone weight.
Loin eye area (LMA): Cross-sectional area of the longissimus dorsi muscle between the 12th and 13th ribs (unit: cm^2^).
Backfat thickness (BFT): Subcutaneous fat thickness over the longissimus dorsi between the 12th and 13th ribs (unit: mm).

### 2.7 Statistical analysis

All data were tested for normality and homogeneity of variance prior to analysis. Data were compiled using Microsoft Excel (Microsoft Corp., Redmond, WA, United States). Two-factor data were analyzed by two-way analysis of variance (ANOVA) using SPSS 21.0 (IBM Corp., Armonk, NY, United States) If a significant interaction or main effect was observed, differences among treatment means at each time point were compared using Duncan’s new multiple range test. The data differences between the two groups in the Yanbian yellow cattle feeding experiment were statistically analyzed using an independent samples *t*-test. Differences were considered statistically significant at *p* < 0.05.

## 3 Results

### 3.1 Optimal ratio of lignocellulolytic enzymes

**FIGURE 1:**
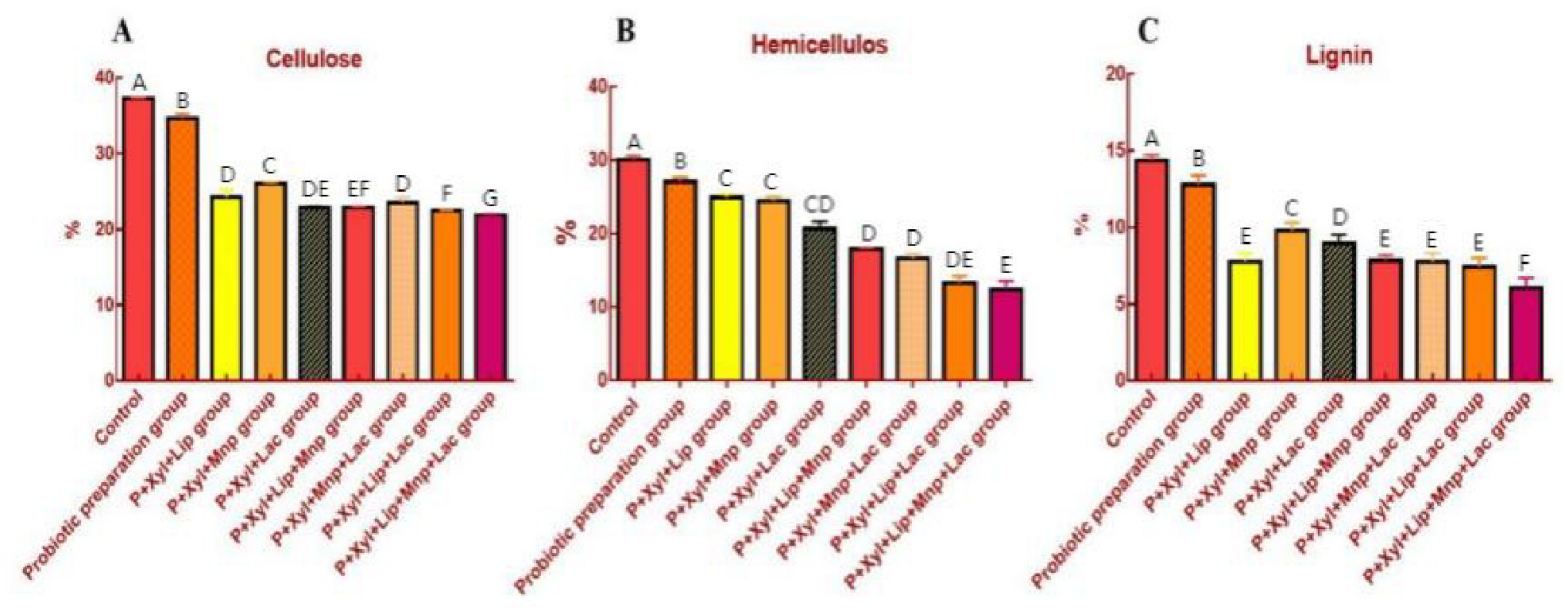
**(A)** Cellulose content in each group. **(B)** Hemicellulose content in each group. **(C)** Lignin content in each group. Different letters (A–G) indicate significant differences among the treatment groups (*P* < 0.01).

As shown in Figures 1A–C, significant differences were observed in cellulose, hemicellulose, and lignin content between the control group and various fermentation groups. Compared to the control, most combined fermentation groups (e.g., the P-Xyl-Lip-Mnp-Lac group) exhibited reductions in cellulose and hemicellulose content, along with alterations in lignin content. These results indicate that Enzyme-Probiotic composite fermentation effectively degrades carbohydrates and lignin in the substrate, with the extent of degradation varying among different combinations. Among them, the ninth group (P-Xyl-Lip-Mnp-Lac) demonstrated the most effective degradation of all three components.

### 3.2 Dynamic changes in structural carbohydrates of corn stover under different treatment conditions during fermentation

#### 3.2.1 Fermentation quality of corn stover

**FIGURE 2:**
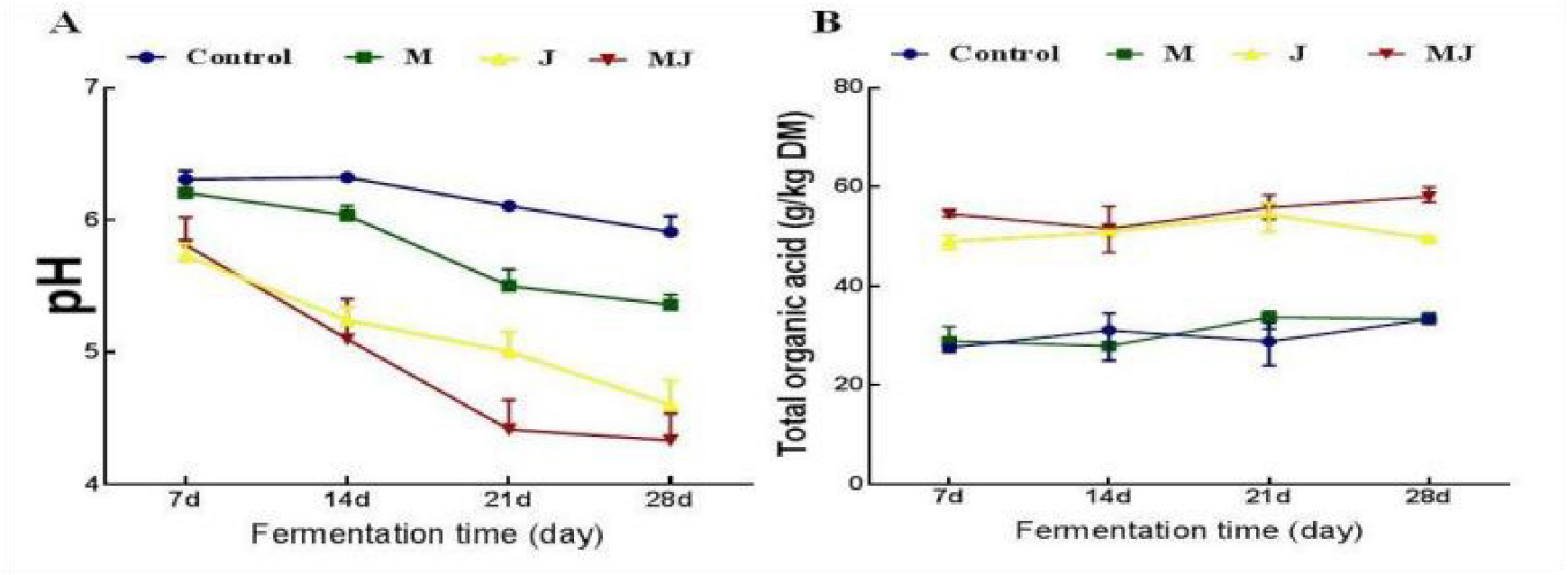
Plots of pH and total organic acids over 28 days of fermentation for different treatments. **(A)** pH variation curve. **(B)** Total organic acid variation curve.

As shown in Table 4, with prolonged fermentation time, the pH and NH3-N levels in the M, J, and MJ groups showed a significant decreasing trend (*p* < 0.05), while the lactic acid (LA) content increased significantly (*p* < 0.05). The pH and NH3-N content in the MJ group were significantly lower than those in the other groups (*p* < 0.05), whereas the lactic acid content was significantly higher compared to the control (CON group) and the M group (*p* < 0.05). Figure 2 illustrates that during the 28-day fermentation period, the pH value decreased rapidly in the MJ and J groups, and the total organic acid content increased significantly compared to the other groups.

**TABLE 4:** Effect of different treatments on the fermentation quality of corn stover (g/kg DM).

Item	Time (day)	Treatments				SEM	*P*-value		
		CON	M	J	MJ		T1	T2	T1 × T2
pH	A1	6.31^Aa^	6.21^Ab^	5.73^Ad^	5.81^Ac^	0.02	< 0.01	<0.01	<0.01
	A2	6.32^Ba^	6.04^Bb^	5.24^Bc^	5.10^Bd^				
	A3	6.11^Ca^	5.50^Cb^	5.01^Cc^	4.42^Cd^				
	A4	5.91^D^	5.36^Db^	4.60^Dc^	4.33^Dd^				
LA (g/kg DM)	A1	23.59^d^	25.45^c^	40.12^b^	43.68^a^	1.85	< 0.01	0.31	< 0.01
	A2	27.23^c^	24.25^d^	42.02^a^	41.90^b^				
	A3	25.14^d^	25.78^c^	46.26^a^	43.70^b^				
	A4	25.37^d^	25.56^c^	39.86^b^	47.20^a^				
AA (g/kg DM)	A1	0.00^Bc^	0.00^Cc^	4.88^Cb^	7.21^Ba^	0.02	< 0.01	<0.01	<0.01
	A2	0.00^Bc^	0.00^Cc^	5.07^Bb^	6.11^Ca^				
	A3	0.00^Bd^	4.43^Ac^	4.69^Db^	8.53^Aa^				
	A4	4.15^Ad^	4.29^Bc^	6.07^Ab^	7.23^Ba^				
PA (g/kg DM)	A1	3.77^Aa^	3.29^Bc^	3.78^Aa^	3.45^b^	0.01	< 0.01	<0.01	<0.01
	A2	3.63^Ba^	3.44^Ab^	3.41^ABb^	3.41^b^				
	A3	3.45^Db^	3.32^ABd^	3.35^Bc^	3.46^a^				
	A4	3.53^Ca^	3.31^ABc^	3.42^ABb^	3.46^b^				
BA (g/kg DM)	A1	0.24^a^	0.21^Cd^	0.22^Ac^	0.22^b^	0.01	< 0.01	<0.01	<0.01
	A2	0.23^c^	0.41^Aa^	0.21^Ad^	0.24^b^				
	A3	0.25^a^	0.21^Cb^	0.18^Bc^	0.21^b^				
	A4	0.22	0.22^B^	0.21^A^	0.22				
NH_3_-N (g/kg DM)	A1	4.24^Aa^	3.89^Ac^	3.94^Ab^	3.74^Ad^	0.09	< 0.01	<0.01	<0.01
	A2	4.05^Ca^	3.63^Bc^	3.77^Bb^	3.65^Bc^				
	A3	4.07^Ca^	3.58^Cc^	3.70^Cb^	3.53^Cc^				
	A4	4.11^Ba^	3.52^Db^	3.50^Dc^	3.14^Dd^				
NH_3_-N/ Total N (%)	A1	4.81^Aa^	4.40^Abc^	4.267^Ac^	4.60^Aab^	0.07	< 0.01	<0.01	<0.01
	A2	4.27^Ba^	3.80^Bb^	3.90^Bb^	3.79^Bb^				
	A3	4.08^Ba^	3.88^Bb^	4.04^Ba^	3.74^Bb^				
	A4	3.86^Ca^	3.90^Ba^	3.46^cb^	3.25^Cc^				

A1, after 7 days of fermentation; A2, after 14 days of fermentation; A3, after 21 days of fermentation; A4, after 28 days of fermentation; DM, dry matter; LA, lactic acid; AA, acetic acid; PA, propionic acid; BA, butyric acid; NH_3_-N, ammonia nitrogen mean values with different superscripts (A–D) in the same column differ within sampling days (*p* < 0.01). Means with different superscripts (a–d) in the same row differ significantly among the treatments (*p* < 0.05). SEM: standard error of the mean. NS, not significant; T1 × T2, *p*-value for time × treatment interaction.

#### 3.2.2 Chemical composition of corn stover

As shown in Table 5, with prolonged fermentation time, the dry matter (DM) content of all groups exhibited a decreasing trend (*p* < 0.05), while the DM content in the MJ group was significantly higher than that in the other groups (*p* < 0.05). The crude protein (CP) content in the MJ and J groups was significantly higher compared to the other groups (*p* < 0.05). In the MJ group, the CP content increased with extended fermentation time. The acid detergent fiber (ADF) and neutral detergent fiber (NDF) contents in the MJ and M groups were significantly lower than those in the CON and J groups (*p* < 0.05). On days 7, 21, and 28 of fermentation, the ash content in the J group was significantly lower than that in the other three groups (*p* < 0.05).

**TABLE 5:** Effect of different treatments on the conventional nutrient composition of corn stover (%).

Item	Time (day)	Treatments				SEM	*P*-value		
		CON	M	J	MJ		T1	T2	T1 × T2
DM	A1	65.40^A^	65.54^A^	65.50^A^	65.38	0.55	<0.01	<0.01	<0.01
	A2	62.37^Bb^	62.32^BC^	64.40^ABa^	63.5^ab^				
	A3	61.54^Bb^	61.47^Bb^	62.32^Bb^	65.45^a^				
	A4	53.19^Cab^	55.86^Cb^	54.85^Cb^	66.64^a^				
CP	A1	5.51^Bb^	5.74^b^	6.04^Aa^	5.85^Ba^	0.01	<0.01	<0.05	<0.01
	A2	5.74^Ab^	5.97^ab^	6.07^Aa^	6.05^Aab^				
	A3	5.95^ABb^	5.74^b^	5.72^Bb^	6.04^Aa^				
	A4	5.59^ABb^	5.80^ab^	5.85^ABab^	6.36^Aa^				
ADF	A1	45.84^a^	33.23^b^	41.47^Aa^	36.15^b^	1.77	<0.01	NS	NS
	A2	43.58	35.33	34.43^B^	34.79				
	A3	43.84^a^	33.83^b^	38.29^ABab^	35.4^b^				
	A4	45.57^a^	34.75^b^	34.00^Bb^	34.3^b^				
NDF	A1	88.49^a^	83.86^b^	82.87^Ab^	72.61^c^	3.42	<0.01	<0.01	<0.01
	A2	89.35^a^	84.38^a^	73.93^Bb^	69.56^b^				
	A3	92.14^a^	83.29^a^	73.9^Bb^	69.41^b^				
	A4	88.58^a^	83.81^b^	71.47^Bc^	66.17^d^				
Ash	A1	7.18^a^	7.21^a^	6.75^b^	6.99^Ba^	0.72	<0.01	<0.01	<0.05
	A2	7.29	7.26	7.10	7.56^A^				
	A3	7.33^bc^	7.58^a^	7.10^bc^	7.48^Aab^				
	A4	7.02^b^	7.42^a^	6.79^b^	7.32^A*Bab*^				
ADL	A1	12.41^A^	9.52^b^	9.25^Ab^	7.62^Ab^	0.82	<0.01	<0.01	<0.05
	A2	12.28^B^	8.83^b^	9.08^A*Bb*^	7.43^A*Bc*^				
	A3	12.11*^C^*	8.27^b^	8.75^Bb^	7.13^Bc^				
	A4	12.01*^C^*	8.04^b^	8.42^Bc^	6.71^*C*d^				

A1, after 7 d of fermentation; A2, after 14 d of fermentation; A3, after 21 d of fermentation;A4, after 28 d. Mean values with different superscripts (A-D) in the same column differ between sampling days (*p* < 0.01). Means with different superscripts (E-H) in the same row differed significantly between treatments (*p* < 0.05). SEM: standard error of the mean. NS, not significant.

#### 3.2.3 Losses of cellulose, hemicellulose, and lignin in corn stover after 28 days of fermentation under different treatments

**FIGURE 3:**
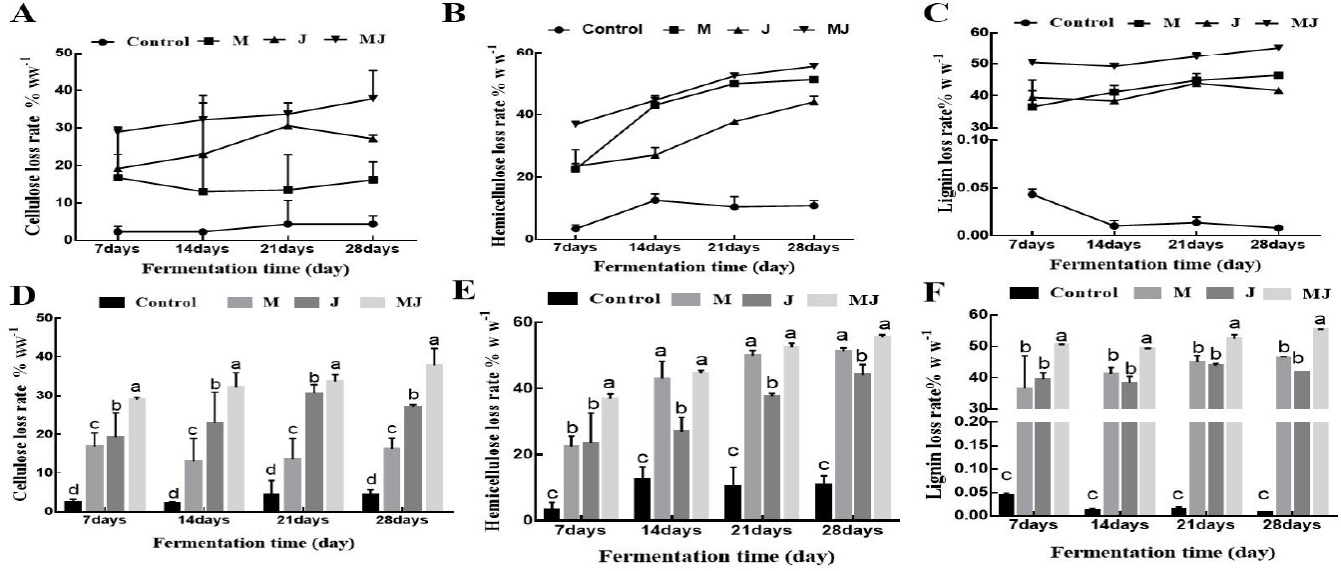
Loss of cellulose, hemicellulose and lignin from corn stover at different treatments for 28 days of fermentation. **(A)** Cellulose loss rate change curve. **(B)** Hemicellulose loss rate change curve. **(C)** Lignin loss rate change curve. **(D)** Cellulose loss rate. **(E)** Hemicellulose loss rate. **(F)** Lignin loss rate. Different letters (a–d) indicate significant differences among the treatment groups (*P* < 0.05).

In the control group, the lignin content remained largely undegraded. After 28 days of fermentation, the losses of cellulose, hemicellulose, and lignin in corn stover (CS) gradually increased across all treatment groups (Figures 3A–C). As shown in Figure 3A, the loss rate of cellulose in CS followed the order: MJ group > J group > M group > control group. The loss rate of hemicellulose exhibited the trend: MJ group > M group > J group > control group (Figure 3B), while the loss rate of lignin decreased in the order: MJ group > M group > J group > control group (Figure 3C).

On days 7, 14, 21, and 28 of fermentation, the losses of cellulose and lignin in the MJ group were significantly greater than those in the M group, J group, and control group (*p* < 0.05) (Figures 3D–F). No significant differences were observed in hemicellulose loss between the MJ and M groups after 14, 21, and 28 days of fermentation (*p* > 0.05). Similarly, there were no significant differences in lignin loss between the M and J groups at the same time points (*p* > 0.05).

#### 3.2.4 Effects of different treatments of fermented corn stover on *in vitro* digestibility

As shown in Table 6, with prolonged fermentation time, the degradation rates of dry matter (DM), crude protein (CP), neutral detergent fiber (NDF), and acid detergent fiber (ADF) in the rumen showed a significant increasing trend (*p* < 0.05) in the control (CON), M, J, and MJ groups. The degradation rates of DM, CP, NDF, and ADF in the rumen were significantly higher in the MJ group compared to the other three groups (CON, M, and J; *p* < 0.05).

**TABLE 6:** Digestive degradation rate of each nutrient after 72 h *in vitro* fermentation of corn stover.

Item	Time (day)	Treatments				SEM	*P*-value		
		CON	M	J	MJ		T1	T2	T1 × T2
DM degradability (%)	A1	29.19^Ac^	35.83^Bb^	31.96^Bc^	46.95^a^	3.18	<0.01	NS	<0.01
	A2	32.32^Ab^	38.42^ABb^	38.08^ABab^	44.52^a^				
	A3	31.80^Ac^	36.61^B*bc*^	41.58^A*Bab*^	44.79^a^				
	A4	23.92^Bb^	42.65^Aa^	44.54^Aa^	48.93^a^				
CP degradability (%)	A1	19.38^Bc^	22.65^B*bc*^	25.07^*C*b^	43.38^a^	0.73	<0.01	<0.01	<0.01
	A2	29.73^Ab^	31.99^Ab^	33.87^Bb^	47.32^a^				
	A3	30.36^Ac^	31.41^Abc^	33.93^Bb^	44.35^a^				
	A4	27.74^Ad^	34.36^Ac^	38.22^Ab^	42.55^a^				
NDF degradability (%)	A1	29.07^b^	33.37^b^	31.71^b^	42.69^a^	0.46	<0.01	NS	NS
	A2	30.90^b^	31.58^b^	31.67^b^	43.04^a^				
	A3	34.51^b^	31.75^b^	33.19^b^	44.15^a^				
	A4	34.27	34.48^b^	34.63^b^	48.03^a^				
ADF degradability (%)	A1	34.07^b^	53.5^a^	40.82^ab^	52.41^a^	2.23	<0.01	NS	NS
	A2	37.39^b^	47.37^a^	45.90^ab^	54.54^a^				
	A3	37.8	46.21	48.86	52.84				
	A4	35.80^b^	52.08^a^	49.89^a^	59.47^a^				

Same as Table 4.

#### 3.2.5 Effects of corn stover on *in vitro* rumen fluid composition

As shown in Table 7, methane production in the MJ group peaked at 28 days of *in vitro* fermentation, while the CON group reached its highest level at day 7. Methane production in the MJ group was significantly lower than that in the other groups at days 7 and 14 (*p* < 0.05). Regarding rumen protozoal counts, the MJ and J groups exhibited significantly higher values compared to the other groups at the same fermentation time points (*p* < 0.05). Fermentation duration had no significant effect on rumen protozoal counts (*p* > 0.05).

**TABLE 7:** Fermentation parameters of corn stover after 72 h of *in vitro* rumen fermentation.

Item	Time (day)	Treatments				SEM	*P*-value		
		CON	M	J	MJ		T1	T2	T1 × T2
pH	A1	6.66^Aa^	5.82^bc^	5.98^Ab^	5.64^c^	0.10	<0.01	<0.01	<0.05
	A2	6.36^B*Ca*^	5.51^c^	5.77^A*Bb*^	5.57^bc^				
	A3	6.47^ABa^	5.617^b^	5.77^A*Bb*^	5.54^b^				
	A4	6.18^*C*a^	5.92^ab^	5.59^B*bc*^	5.31^c^				
NH_3_-N (mg/dL)	A1	13.54^*C*b^	21.84^a^	20.54^Ba^	23.45^*C*a^	0.63	<0.01	< 0.01	NS
	A2	15.18^Bd^	25.8^c^	23.08^Ab^	24.34^*C*a^				
	A3	18.15^Ab^	24.80^a^	24.55^Aa^	26.08^Ba^				
	A4	19.97^Ab^	25.20^a^	25.19^Aa^	28.03^Aa^				
LA (mg/dL)	A1	1.44^d^	2.412^Dc^	3.83^b^	4.61^a^	0.13	<0.01	NS	<0.01
	A2	1.26^d^	2.57^*C*c^	3.47^b^	4.69^a^				
	A3	1.47^d^	2.74^A*Bc*^	3.60^b^	4.36^a^				
	A4	1.52^d^	3.02^Ac^	3.54^b^	4.45^a^				
Number of rumen protoworms × 10^5^/mL	A1	1.89^a^	1.79^b^	1.85^a^	1.89^a^	0.01	<0.01	0.14	0.17
	A2	1.78^b^	1.81^ab^	1.88^ab^	1.91^a^				
	A3	1.82^b^	1.88^ab^	2.06^a^	2.04^a^				
	A4	2.02^ab^	1.87^b^	2.18^a^	2.31^a^				

LA, lactic acid; NH_3_-N, ammonia nitrogen; same as Table 4.

As presented in Table 8, the total volatile fatty acid (TVFA) and NH3-N contents increased significantly with prolonged fermentation time in all treatment groups (*p* < 0.05). The pH value in the MJ group was significantly lower than that in the CON, M, and J groups (*p* < 0.05). Furthermore, the contents of acetic acid (AA), lactic acid (LA), NH3-N, and TVFA in the MJ group were significantly higher than those in the other groups (*p* < 0.05). Butyric acid and isobutyric acid contents in the MJ, M, and J groups were significantly lower than those in the control group (*p* < 0.05). No significant differences were observed in valeric acid and isovaleric acid contents among all treatment groups. Meanwhile, the acetate-to-propionate ratio in the MJ treatment group was significantly higher than that in the other groups at days 7 and 28 (*p* < 0.05).

**TABLE 8:** Total volatile fatty acid content and proportion of various volatile fatty acids in corn stover after 72 h of *in vitro* fermentation.

Item	Time (day)	Treatments				SEM	*P*-value		
		CON	M	J	MJ		T1	T2	T1 × T2
TVFA mmol/L	A1	45.95^c^	52.93^Bc^	63.03^b^	74.93^Ca^	0.92	<0.01	< 0.01	NS
	A2	49.23^c^	60.03^ABbc^	67.88^b^	85.47^BCa^				
	A3	48.66^c^	66.82^Ab^	69.62^b^	94.76^ABa^				
	A4	53.22^d^	69.11^Ab^	77.63^b^	97.99^Aa^				
Acetic acid, %	A1	56.91^b^	59.93^Cb^	71.42^a^	70.78^Ba^	6.15	<0.01	<0.01	<0.01
	A2	61.90	63.36^AB^	68.08	68.31^C^				
	A3	60.88^c^	61.15^BCc^	65.88^b^	72.11^Ba^				
	A4	56.19^c^	65.13^Ab^	63.16^b^	76.64^Aa^				
Propionic acid, %	A1	22.08^a^	20.68^a^	19.11^Cb^	22.02^Aa^	2.40	<0.01	<0.01	<0.01
	A2	18.11	19.27	19.44^C^	19.15^B^				
	A3	19.22	21.02	22.37^BC^	19.66^B^				
	A4	23.29^a^	19.46^b^	24.58^Aa^	19.70^Bb^				
Isobutyric acid, %	A1	2.08^a^	1.79^b^	2.00^b^	1.84^b^	0.33	<0.01	NS	NS
	A2	2.08^a^	1.70^b^	1.57^b^	2.04^b^				
	A3	2.13^a^	1.46^b^	1.55^b^	1.85^b^				
	A4	2.23^a^	1.75^b^	1.46^b^	1.35^b^				
Butyric acid, %	A1	11.89^a^	10.83^b^	10.95^b^	11.70^b^	0.84	<0.05	NS	NS
	A2	11.94^a^	11.06^b^	11.02^b^	11.55^b^				
	A3	11.81^a^	10.59^b^	11.24^b^	9.87^b^				
	A4	11.99^a^	10.56^b^	11.05^b^	9.58^b^				
Valeric acid, %	A1	2.36	2.30	2.20	2.38	0.37	NS	NS	NS
	A2	2.64	2.26	2.03	2.35				
	A3	2.48	2.24	1.86	2.01				
	A4	2.14	2.22	2.16	2.07				
Isovaleric acid, %	A1	2.05	2.06	1.63	1.87	0.34	NS	NS	NS
	A2	1.72	1.88	1.91	2.13				
	A3	1.84	1.86	2.11	1.73				
	A4	1.91	1.64	1.99	1.73				
Acetic acid/propionic acid	A1	2.61^c^	2.90^bc^	3.74^Aa^	3.22^Cb^	0.53	<0.01	<0.01	<0.01
	A2	3.58	3.30	3.50^A^	3.57^B^				
	A3	3.19	2.97	2.96^B^	3.67^B^				
	A4	2.43^c^	3.35^b^	2.58^Bc^	3.89^Aa^				

TVFA, Total VFA concentration; same as Table 4.

### 3.3 Fermentation characteristics and correlation with fermentation parameters of corn stover

#### 3.3.1 Effects of different treatments on fermentation quality and conventional nutrient composition of corn stover

**FIGURE 4:**
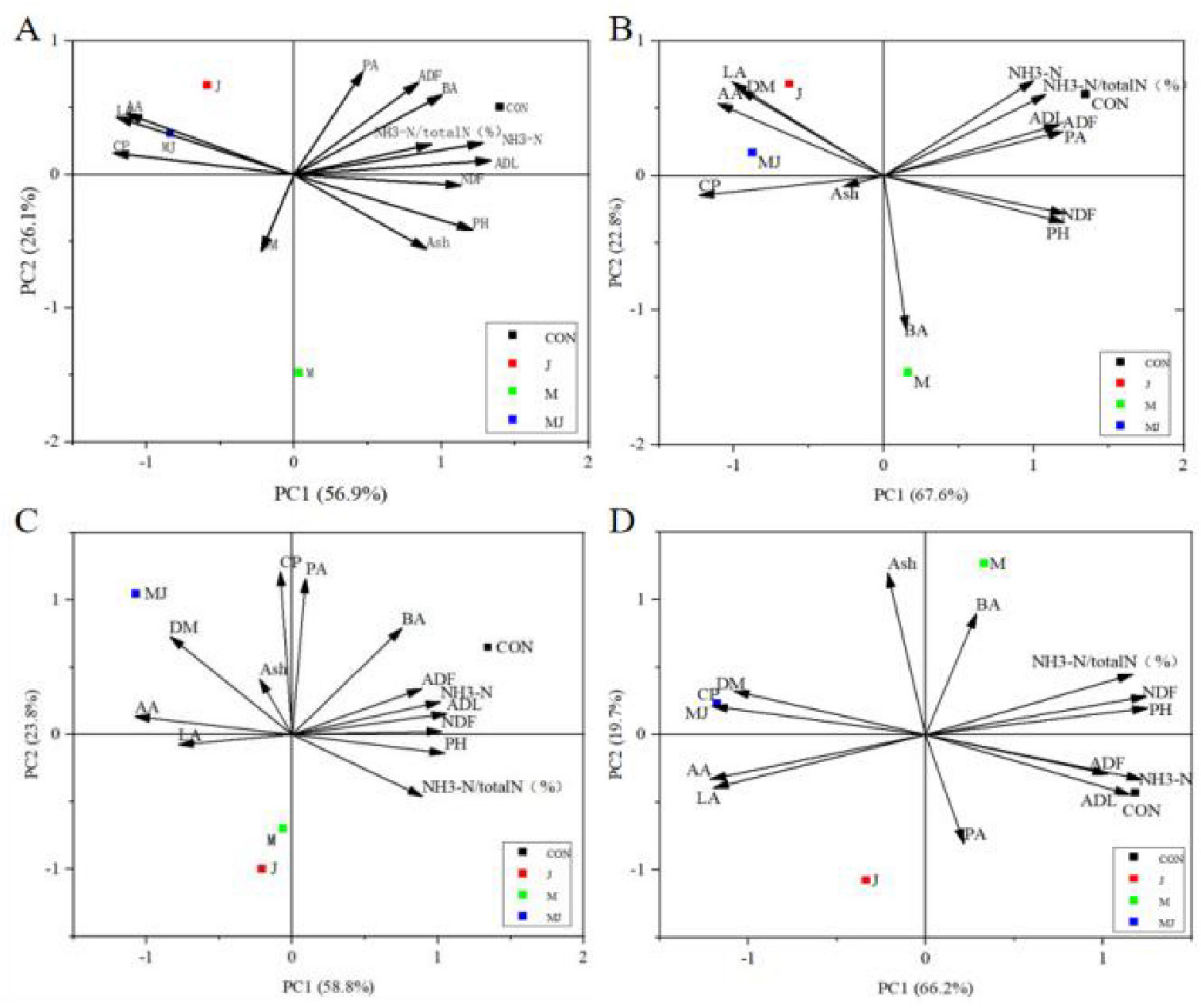
Biplot of principal component analysis (PCA) illustrating the association between fermentation characteristics and fermentation days (7, 14, 21, and 28). CON, control group; M, cellulase group; J, compound probiotics group; MJ, compound probiotics + cellulase group; LA, lactic acid; AA, acetic acid; PA, propionic acid; NH3-N, ammonia nitrogen; NH3-N/total N, ratio of ammonia nitrogen to total nitrogen; DM, dry matter; CP, crude protein; ADF, acid detergent fiber; NDF, neutral detergent fiber; Ash, ash content; ADL, acid detergent lignin. **(A)** Day 7 of fermentation; **(B)** day 14 of fermentation; **(C)** day 21 of fermentation; **(D)** day 28 of fermentation.

Principal component analysis (PCA) is presented in Figure 4. Distinct separation was observed among the CON, M, J, and MJ groups at day 28. As shown in the figure, the correlations between fermentation characteristics and treatment groups exhibited dynamic temporal patterns: at day 7, NH3-N, ADF, ADL, BA, and PA showed positive correlations with the CON group, while LA, AA, and CP correlated positively with the M and MJ groups; by day 14, NH3-N, NH3-N/total N, ADF, ADL, and PA maintained positive correlations with CON, whereas LA, AA, DM, CP, and Ash were positively correlated with the M and MJ groups; at day 21, ADF, ADL, and NH3-N/total N remained positively correlated with CON, while CP and PA showed negative correlations with J and M groups, and DM, LA, AA, CP, and PA exhibited positive correlations with MJ group; by day 28, ADF and ADL continued to show positive correlations with CON, pH was negatively correlated with J group, Ash was positively correlated with M group, and DM, CP, LA, and AA maintained positive correlations with MJ group.

#### 3.3.2 Digestibility of nutrients in corn stover after 72 h of *in vitro* fermentation

**FIGURE 5:**
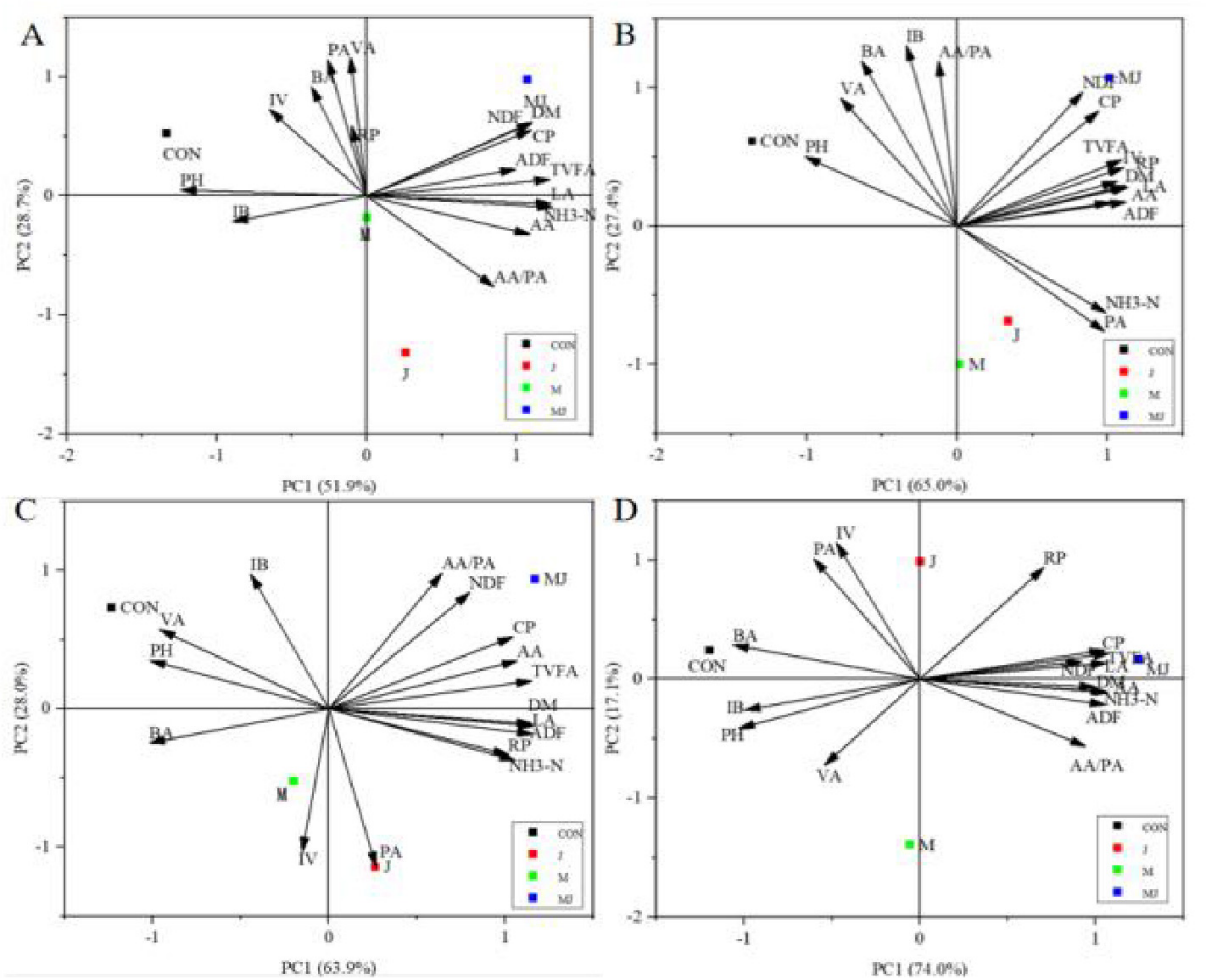
Principal component analysis (PCA) biplot showing the relationships between fermentation characteristics and fermentation time points (7, 14, 21, and 28 days). CON, control group; M, cellulase group; J, compound probiotics group; MJ, compound probiotics + cellulase group; DM, dry matter; CP, crude protein; NDF, neutral detergent fiber; ADF, acid detergent fiber; NH3-N, ammonia nitrogen; RP, rumen protozoa; TVFA, total volatile fatty acids; AA, acetic acid; LA, lactic acid; PA, propionic acid; IB, isobutyric acid; BA, butyric acid; VA, valeric acid; IV, isovaleric acid; AA/PA, acetic acid to propionic acid ratio. **(A)** Day 7; **(B)** day 14 **(C)** day 21; **(D)** day 28.

Principal component analysis (PCA) is presented in Figure 5. Distinct separation was observed among the CON, M, J, and MJ groups at day 28. As illustrated in the figure, the associations between treatment groups and fermentation characteristics exhibited dynamic temporal patterns: at day 7, pH showed positive correlation with the CON group while NDF, DM, CP, ADF, TVFA, LA, NH3-N, and AA demonstrated positive correlations with the MJ group, with LA, NH3-N, and AA also correlating positively with the J group; by day 14, pH maintained positive correlation with the CON group while showing negative correlation with NH3-N, with TVFA, NDF, and CP preserving positive correlations with the MJ group whereas branched-chain fatty acid indicators (BA and IB) exhibited negative correlations with both J and M groups; at day 21, pH remained positively correlated with the CON group while IV and PA showed positive correlations with the J group and IB demonstrated negative correlation with the M group, alongside TVFA, AA/PA ratio and NDF displaying positive correlations with the MJ group; through day 28, PA and IV sustained positive correlations with the J group while the M group displayed minimal associations with most parameters, and TVFA, CP and LA maintained positive correlations with the MJ group whereas BA and IB showed positive correlations with the CON group.

### 3.4 effects of enzyme-probiotic complex fermented feed on growth performance and slaughter traits of Yanbian yellow cattle

As shown in Table 9, the final body weight of the experimental group was significantly higher than that of the control group (*p* < 0.05). The average daily gain was significantly higher than that of the control group (*p* < 0.01), and the feed-to-gain ratio was significantly lower than that of the control group (*p* < 0.01).

**TABLE 9:** Effects of feeding fermented feed with probiotic-enzyme complex on growth performance of Yanbian yellow cattle.

Item	Unit	CON (*n* = 12)	FER (*n* = 12)	*P*
IBW	kg	450.2 ± 15.8	452.5 ± 14.3	> 0.05
FBW	kg	580.7 ± 22.1	605.4 ± 19.5*	< 0.05
ADG	kg/天	1.45 ± 0.08	1.70 ± 0.07**	< 0.01
ADFI	kg DM/头/天	8.95 ± 0.35	9.10 ± 0.32	> 0.05
F/G	kg DM/kg	6.17 ± 0.25	5.35 ± 0.18**	< 0.01

*P < 0.05 (significant difference compared with the control group); **P < 0.01 (highly significant difference compared with the control group); n = number of cattle; data are expressed as mean ± standard deviation (Mean ± SD).

As shown in Table 10, the slaughter body weight, carcass weight, dressing percentage, meat-to-carcass ratio, and backfat thickness at the 12th–13th rib interface of the experimental group were significantly higher than those of the control group (*p* < 0.05), while the net meat weight, net meat percentage, loin eye area, and meat-to-bone ratio were extremely significantly higher compared to the control group (*p* < 0.01).

**TABLE 10:** Effects of feeding probiotic-enzyme complex fermented feed on slaughter performance of Yanbian yellow cattle.

Item	Unit	CON (*n* = 6)	FER (*n* = 6)	*P*
SBW	kg	575.3 ± 20.5	600.8 ± 18.7*	< 0.05
CW	kg	322.5 ± 12.8	342.1 ± 11.6*	< 0.05
DR	%	56.05 ± 0.85	56.92 ± 0.78*	< 0.05
NMW	kg	265.8 ± 11.2	285.3 ± 10.5**	< 0.01
NMR	%	46.18 ± 0.92	47.48 ± 0.86**	< 0.01
Meat-to-carcass ratio	%	82.42 ± 1.05	83.41 ± 0.98*	< 0.05
LMA	cm^2^	78.6 ± 3.2	85.4 ± 3.5**	< 0.01
BFT	mm	12.5 ± 1.8	10.2 ± 1.5*	< 0.05
Meat-to-bone ratio	–	4.85 ± 0.21	5.22 ± 0.19**	< 0.01

*P < 0.05 (significant difference compared with the control group). **P < 0.01 (highly significant difference compared with the control group); n = number of cattle; data are expressed as mean ± standard deviation (Mean ± SD).

## 4 Discussion

### 4.1 Synergistic degradation mechanism of the enzyme-microbe composite system and advantages in fermentation initiation

The enzyme-microbe composite system (MJ group) constructed in this study demonstrates significant advantages in enhancing the fermentation quality and feed value of corn stover. The core of this lies in the rational design and synergistic action of the composite enzyme system and functionally complementary microbial communities.

Lignocellulose, composed of cellulose, hemicellulose, and lignin, is an essential structural component of plant cell walls that limits microbial access to fermentable substrates (Chen et al., 2025). To overcome this bottleneck, we systematically compared and optimized various enzyme formulations, microbial concentrations, and treatment methods in preliminary experiments. By comprehensively analyzing enzymatic hydrolysis efficiency and product yield, we ultimately identified the most suitable enzyme combination scheme, which involves using a total enzyme concentration of 0.2% (w/w), with cellulase, xylanase, lignin peroxidase, manganese peroxidase, and laccase mixed in a ratio of 1:1:1:1:1. This combination demonstrated a synergistic effect in the treatment of lignocellulose, significantly enhancing substrate accessibility and degradation efficiency. This study showed that the quality of corn stover fermented with the enzyme-microbe synergy was superior to that fermented with either microbial or enzymatic preparations alone. Previous research indicates that the addition of cellulase can enhance the hydrolysis and release of fermentable sugars, providing sufficient substrates for *lactic acid bacteria* (Du et al., 2024). As lactic acid is produced and pH drops, acid-sensitive anaerobic microbes disappear, allowing *lactic acid bacteria* to proliferate rapidly. Their metabolic activity results in substantial synthesis and accumulation of lactic acid, with beneficial bacteria such as *Lactobacillus* increasingly dominating the microbial community. This succession of microbial populations significantly improves stover fermentation quality (He et al., 2025), consistent with the findings of this study. The pH of corn stover in the enzyme-microbe composite fermentation was significantly lower than that of the single-enzyme group on days 7, 14, 21, and 28, indicating that the exogenous addition of *lactic acid bacteria* can promote this ecological succession and, thereby, enhance fermentation quality. As heterofermentative bacteria, *lactic acid bacteria* can ferment hexose carbohydrates into lactic acid via glycolysis and pentose sugars into lactic acid and acetic acid via the phosphoketolase pathway (Ventimiglia et al., 2015), which explains why acetic acid levels in the MJ and J groups were significantly higher than in the M and CON groups. Moreover, studies have shown that lactic acid to acetic acid-dominated fermentation can improve the preservation of corn biomass and promote *lactic acid bacteria* proliferation (Okoye et al., 2025), further enhancing corn stover fermentation.

Corn stalks are the waste products of corn plants left in the field after the harvest of corn cobs. Among the many types of corn residues produced, corn stalks are seldom utilized. The hemicellulose contained in them is the second most abundant component of lignocellulosic agricultural residues and is primarily composed of xylan. Exogenously added xylanase can specifically act on the xylan components in the cell walls of the stalks, producing soluble xylo-oligosaccharides (RX) and xylo-oligosaccharide fragments rich in xylobiose and xylotriose (XOS) through hydrolysis (Zhao et al., 2025). XOS serves as a carbon source for *Lactobacillus*, promoting its proliferation and leading to a significant accumulation of lactic acid (Bian et al., 2013), which causes the pH of the fermentation system to drop rapidly and inhibits the growth of harmful bacteria. Yeast exhibits a synergistic fermentation function with *Lactobacillus*, possessing both acid tolerance (Zhang et al., 2025) and biotransformation capabilities, allowing it to convert pentose sugars and other monosaccharides into ethanol (da Silva Fernandes et al., 2022). As fermentation progresses, ethanol continues to accumulate, working synergistically with the acidic environment formed earlier to produce a significant antimicrobial effect, collectively establishing a robust “acid-ethanol” composite barrier. This barrier not only effectively suppresses the invasion and growth of contaminating microorganisms but also ensures the long-term stable storage of fermented corn stalks.

*Bacillus subtilis* is a member of the Bacillus genus, known for its strong resilience and ability to secrete various enzymes, and is widely used in the fermentation of agricultural and sideline products to enhance their nutritional value (Gao et al., 2025). Research results indicate that the rates of cellulose and hemicellulose loss in the enzyme-probiotic composite fermentation group are higher than those in the single-enzyme addition group, suggesting that the composite enzyme-probiotic preparation promotes the breakdown of both, which may be related to the enzymes secreted by *Bacillus subtilis*. Hou et al. demonstrated that adding *Bacillus subtilis* can intensify cellulose degradation and disrupt the structure of hemicellulose in cell walls during the fermentation process (Hou et al., 2025), which is consistent with the findings of this study. The CP values of groups J and MJ were significantly higher than those of group M, which may be associated with a reduction in protease abundance in silage following *Bacillus subtilis* inoculation (Bai et al., 2022). Additionally, *Bacillus subtilis*-mediated enhancement of acetolactate synthase activity can catalyze the conversion of pyruvate to acetolactate, thereby improving the nutritional value and palatability of fermented feed (Bonaldi et al., 2021).

The enzyme-microbe composite system developed in this study significantly enhances fermentation quality primarily due to the formation of a progressively amplified positive feedback loop within the system. This loop begins with the initial highly efficient hydrolysis by the composite enzymes, providing *lactic acid bacteria*, yeast, and *Bacillus subtilis* with abundant fermentation substrates. It subsequently drives *lactic acid bacteria* and yeast to rapidly establish an “acid-alcohol” barrier, while *Bacillus subtilis* further intensifies the degradation of fibrous structures by secreting a variety of auxiliary enzymes. This optimized environment, in turn, ensures and enhances the sustained activity of the entire microbial community and enzymatic system, thereby promoting the more thorough breakdown of lignocellulose and achieving a self-propelling and continuously optimized fermentation process.

### 4.2 Enhancement of nutritional value of fermentation products and rumen digestive kinetics

The addition of enzyme preparations to corn straw releases a substantial amount of soluble sugars, providing abundant fermentable substrates for the growth of microorganisms such as *lactic acid bacteria*. This promotes the production of lactic acid and volatile fatty acids, creating an acidic environment that inhibits the growth of harmful microorganisms and reduces NH3-N content, thereby decreasing the loss of protein and nutrients in silage (Broderick and Kang, 1980). This is a key reason why the CP content in the J and MJ groups was significantly higher than in the other groups and why the DM loss in the MJ group was significantly lower than in the other groups. In our study, the ADF and NDF in the MJ group decreased significantly, likely due to the interaction between *Bacillus subtilis*, *Lactobacillus*, and the composite enzyme preparation. The lignocellulose-degrading enzyme system is crucial for initiating cellulose degradation; it breaks down stubborn cellulose chains to generate fermentable sugars, providing essential substrates for the colonization and proliferation of *Bacillus subtilis* and *Lactobacillus plantarum*. Furthermore, these two functional strains are not merely passive consumers of substrates; the auxiliary cellulases they secrete during metabolism can complement the exogenous enzyme system, thereby enhancing the cellulose degradation process. This bidirectional interaction between enzymes and microbes jointly drives the efficient conversion of cellulose into usable nutrients (Su et al., 2024). Li et al. also found that co-fermentation with *lactic acid bacteria* and cellulases can more effectively decompose insoluble sugars. These findings indicate that enzyme-microbe fermentation can effectively reduce lignocellulose content and improve the nutritional value of fermented corn straw (Ma et al., 2025).

The ruminal digestion rates of DM and NDF in straw are key factors determining livestock DMI, and thus are important indicators for evaluating nutritional value (Nuez-Ortín and Yu, 2010). The results of this study indicate that corn straw treated with enzyme-microbial compound fermentation (MJ group) exhibited significantly higher *in vitro* ruminal digestibility of DM, NDF, and ADF compared to other treatment groups. Research by Cao et al. suggests that enzymatic degradation of lignocellulose loosens the connections between cellulose and hemicellulose (Cao et al., 2011), thereby facilitating the attachment and colonization of rumen microbes on the lignocellulose surface, which helps improve NDF and ADF digestibility, consistent with the findings of this study. Additionally, the introduction of probiotics and enzymes promoted the disruption of plant cell wall structures, leading to the rapid release of intracellular contents. This, in turn, provides rumen microbes with more fermentable substrates, ultimately enhancing ruminal degradation (Chen et al., 2016; Li et al., 2017).

During rumen fermentation, the process of methane generation results in the expulsion of carbon (C) and hydrogen (H) from the feed that could otherwise be utilized by the animal, in the form of CH_4_. This chemical process inherently consumes a substantial amount of metabolic energy. When rumen fermentation reaches the peak of methane production, it signifies energy loss and reduced feed efficiency (Antonius et al., 2024; Hnokaew et al., 2025). Moreover, as CH4 is one of the most potent greenhouse gases, mitigating its emissions is crucial for sustainable development. The use of probiotics and other nutritional strategies to regulate the rumen microbiota has become a research hotspot. Scholars have explored various nutritional and microbial approaches, and probiotics are gaining attention due to their natural origin and environmentally friendly characteristics (Saleem et al., 2025b). Among them, *Lactobacillus* and yeast have been shown to have the potential to modulate fermentation in ruminants, improve feed conversion efficiency, and alter microbial populations to reduce methane production (Saleem et al., 2025a). In contrast, by day 28, the MJ group reached its peak methane content, whereas the CON group had already peaked by day 7. This phenomenon indicates that while the enzyme-microbe composite treatment did not completely suppress methane production, it significantly altered the fermentation pattern, delaying the methane production peak and associating it with higher fiber degradation and a longer nutrient utilization cycle. This provides a new perspective on regulating the fermentation process through biological pretreatment, reducing methane emission intensity while maintaining digestibility.

Volatile fatty acids (VFAs) are key indicators of rumen feed fermentation capacity. As the main energy source, short-chain fatty acids (SCFAs) provide more than 70% of the metabolizable energy supply for ruminants. Research by Liu et al. indicates that the addition of probiotics may promote the production of SCFAs in the rumen (Li and Song, 2022) and improve net energy utilization. Propionate serves as a precursor for glucose synthesis, which supplies most of the energy required by organisms. The substantial breakdown of propionate observed on day 28 suggests that the fermentation system has entered a stage of more active energy metabolism and utilization. At this point, readily degradable fiber components have been largely consumed, and rumen microbes shift their metabolic focus to utilizing precursors such as propionate for gluconeogenesis to maintain a continuous energy supply. This phenomenon indirectly confirms that, through long-term enzyme-probiotic synergistic fermentation, straw has been transformed into a form of energy more efficiently utilized by microbes, thereby driving more active bodily metabolism. The increase in total short-chain fatty acid content can serve as an indicator of improved carbohydrate fermentation activity in the rumen. Interestingly, the pH value in the MJ group did not decrease significantly, indicating that the rumen exhibited effective buffering (Belanche et al., 2023), which aligns with the findings of Alejandro et al. In the MJ group, the fiber structure of corn straw was effectively disrupted, releasing the encapsulated proteins, allowing proteins that were originally difficult for rumen microbes to access and utilize to be decomposed and utilized by microbes, generating more NH3-N. Simultaneously, the increased total short-chain fatty acid content and protozoa abundance in the MJ group also suggest that the rumen micro-ecosystem is in a highly efficient and vigorous metabolic state.

### 4.3 Interaction effect of *in vitro* fermentation time and treatment method

Principal Component Analysis (PCA) illustrated the correlation between fermentation parameters and treatments in silage at 7–28 days and after 72 h. In each plot, correlations are indicated by the angles between arrows pointing to two variables: acute angles represent positive correlations, right angles indicate no correlation, and obtuse angles indicate negative correlations (de Melo et al., 2023). This study, through PCA, revealed that during the 7–28 day fermentation process, the combined supplementation of compound probiotics and lignocellulose enzymes (MJ group) demonstrated significant and consistent synergistic advantages in terms of fermentation quality, nutrient preservation, and fiber degradation compared to single treatment groups and the control. This advantage is not a mere additive effect of single treatments but represents a dynamic and systemic improvement throughout the fermentation process. In the early fermentation stage (7 days), the MJ group was closely associated with nutritional indicators (DM, CP), indicating that the combined treatment can rapidly establish a microecological environment conducive to nutrient preservation. Probiotics may preferentially utilize soluble carbohydrates for growth and acid production, quickly lowering pH and thereby inhibiting premature activity of protein-degrading bacteria. As fermentation progressed to 14 days, the correlation between the MJ group and lactic acid, acetic acid, and other products continued to strengthen, suggesting that its acid-producing capacity is not only rapidly initiated but also sustainably enhanced. This creates a beneficial “acid production-inhibition-stability” cycle.

Crucially, the MJ group maintained a comprehensive advantage even on day 28 of fermentation, whereas metabolic activity in the J group weakened after 72 h. This strongly suggests that the soluble sugars produced by lignocellulolytic enzymes breaking down fibers provide a continuous substrate for probiotics, preventing their decline due to the depletion of their own nutrients, thereby significantly enhancing the stability of the fermentation system. In stark contrast, from day 7 to 28 of fermentation, the CON group remained consistently associated with pH and ammoniacal nitrogen. Ammoniacal nitrogen is the end product of protein decomposition by microorganisms, and its accumulation often directly reflects excessive protein degradation and energy loss. The low ammoniacal nitrogen in the MJ group indicates that its lower pH effectively inhibited the activity of spoilage bacteria and ammonia-producing bacteria, thereby maximizing the protection of crude protein.

### 4.4 The final impact on the production performance of ruminants

Significant improvements in *in vitro* fermentation quality ultimately aim to translate into effective enhancements in animal production performance. Therefore, exploring the potential effects of MJ treatment on the growth and production efficiency of ruminants is an essential step in evaluating its application value.

Slaughter performance is an important indicator for effectively demonstrating the economic profitability of raising cattle. When assessing slaughter performance, carcass weight and dressing percentage are among the key indicators. Research has shown that the energy content of feed can influence carcass weight—the higher the feed energy content, the heavier the carcass (Gökdal et al., 2012; Ma et al., 2019). In this study, the carcass weight and dressing percentage of the MJ group were significantly higher than those of the control group. This may be attributed to the higher short-chain fatty acid content in corn stover after enzyme-microbe co-fermentation. Moreover, with the national slaughter rate in China at 52% and price fluctuations of 0.3 yuan/kg (Zhou et al., 2001), the dressing percentage of Yanbian yellow cattle fed the MJ-treated feed in this study reached 56.92%, indicating that feeding enzyme-microbe fermented corn stover helps improve economic efficiency in livestock production.

Studies have shown that increased energy intake concentrations can increase backfat thickness and eye muscle area (Kang et al., 2020), aligning with our findings of a significant increase in eye muscle area and 12–13th rib backfat thickness. When energy supply is adequate, ingested protein can be more efficiently used for body protein (muscle) synthesis rather than passively broken down as an energy source, which collectively leads to a substantial increase in net meat yield and meat-to-bone ratio. Similarly, under sufficient energy supply, dietary protein can be utilized more efficiently for body protein synthesis rather than as an energy source, further contributing to the significant improvement in net meat yield and meat-to-bone ratio.

In summary, MJ treatment, by improving feed energy supply and protein utilization efficiency, comprehensively optimizes ruminant slaughter performance and carcass quality, providing strong evidence for its practical application in production. Although this study did not conduct *in vivo* digestion experiments to obtain direct digestion rate data, the improvement in fermentation indicators was ultimately effectively validated by the animal meat production performance, demonstrating the practical value of this treatment.

### 4.5 Feasibility, scalability, and economic analysis of fermentation products

The fermentation products of the MJ group demonstrate significant advantages in terms of technical feasibility, primarily due to the synergistic production of high-quality and highly stable fermentation products. Higher levels of lactic acid and acetic acid, along with lower pH and ammoniacal nitrogen, indicate excellent acid preservation effects, effectively inhibiting harmful microorganisms and extending shelf life (Muglia et al., 2025). Improved crude protein retention and fiber degradation capacity directly enhance the digestibility and feed value of the products.

Both compound probiotics and enzyme preparations are common feed additives, and their mode of addition (mixed spraying) can be easily integrated into existing solid-state fermentation or silage production lines without major modifications to core equipment. The fermentation stability demonstrated by the MJ group suggests lower process variability in large-scale production, making it easier to achieve product consistency and standardization, which is key to successful expansion.

The incremental costs of this fermentation strategy mainly come from externally added compound probiotics and lignocellulosic enzyme preparations. However, the benefits include higher crude protein retention, which translates to savings on expensive protein feed ingredients; soluble sugars and energy (volatile fatty acids) from cellulose decomposition, which enhance the overall nutritional value of the product and reduce per-unit feed costs for animals; and excellent fermentation stability, which lowers the economic risks of batch spoilage. Overall, although the MJ strategy introduces additional costs for microbial agents and enzymes, the comprehensive economic benefits achieved through significant improvements in product quality and stability are likely to offset and even exceed these incremental costs, demonstrating strong technical and economic feasibility and industrial application potential. Future research may focus on precisely quantifying these benefits in large-scale production for more accurate cost-benefit analysis.

## 5 Conclusion

The conclusions of this study indicate that enzyme-microbe composite treatment (MJ group) can synergistically promote the effective degradation of lignocellulosic structures in corn stalks, significantly improving their fermentation quality, as evidenced by a marked increase in lactic acid content and a notable decrease in pH and ammonia nitrogen levels. This treatment not only enhances the *in vitro* ruminal digestibility and volatile fatty acid production of the stalks but also effectively optimizes rumen fermentation patterns, delaying the peak of methane emissions and improving energy utilization efficiency. Ultimately, these positive physicochemical and microbiological effects are successfully translated into substantial improvements in livestock production performance, significantly increasing the daily weight gain, feed conversion ratio, and slaughter performance of Yanbian yellow cattle. Therefore, enzyme-microbe synergistic treatment represents an effective strategy to comprehensively enhance the feed value of corn stalks and boost ruminant production performance, providing a reliable technical pathway for the resource utilization of agricultural by-products.

## Data Availability

The data reported in this manuscript have been deposited in the OMIX, China National Center for Bioinformation under accession number OMIX013215, https://ngdc.cncb.ac.cn/omix/.
